# Acute or Short-term Effects of Whey Protein Alone or Along with Carbohydrate on Inflammation: A Systematic Review of Clinical Trials -

**DOI:** 10.31661/gmj.v12i.2441

**Published:** 2023-04-18

**Authors:** Ali Akbari, Mahsa Moazen, Siavash Babajafari, Seyedeh Maryam Abdollahzadeh, Maryam Ranjbar Zahedani, Najmeh Sasani, Asma Kazemi

**Affiliations:** ^1^ Department of Anesthesiology, School of Medicine, Shiraz University of Medical Sciences, Shiraz, Iran; ^2^ Nutrition Research Center, School of Nutrition and Food Sciences, Shiraz University of Medical Sciences, Shiraz, Iran; ^3^ Department of Nutrition Sciences, School of Health, Larestan University of Medical Sciences, Larestan, Iran

**Keywords:** Inflammation, Whey Proteins, Carbohydrates, Acute, Systematic Review

## Abstract

Background: Excessive inflammatory response is associated with several diseases. Recently, there has been an increasing trend for investigation of the acute or short-term effects of whey protein alone or in combination with carbohydrates on inflammatory status, especially in athletes. This systematic review aimed to clarify these effects.Materials and Methods: PubMed, Scopus, and Web of Science databases were searched from January 1990 to September 2021, without language restriction. Adult studies examining the effects of whey protein alone or together with carbohydrates on interleukin-6, tumor necrosis factor-Î±, and C-reactive protein levels with a maximum duration of 15 days and with at least one comparison group were included. The quality of studies was analyzed using the Cochrane risk of bias tool.Results: Twenty-five studies met the inclusion criteria. Significant reductions in inflammatory markers was observed in seven out of 25 studies (28%). However, one out of 25 studies (4%) reported a significant increase in inflammatory status. Among those studies comparing the effects of whey protein alone with non-protein or protein-containing groups, 18.18% (two out of 11) and 10% (one out of ten) of the trials revealed a significant decrease in the markers, respectively. Moreover, of those studies comparing whey protein plus carbohydrate with non-protein or protein-containing groups, 33.33% (two out of six) and 40% (two out of five) of them showed a significant reduction in the inflammatory response, respectively. The quality of the majority of studies (84%) was poor.Conclusion: It seems that whey protein alone or the combination of it with carbohydrates may not affect the inflammatory markers in the short run (PROSPERO registration number: CRD42021273915).[GMJ.2023;12:e2441]

## Introduction

Inflammation is one of the major processes involved in the body’s defense against injuries or infections [1]. However, excessive inflammatory response and cytokine overproduction can result in the development of several diseases including rheumatoid arthritis, atherosclerosis, diabetes, obesity, Alzheimer’s disease, and multiple sclerosis [2][3]. Inflammatory diseases can lead to high rates of disability and mortality around the world, accounting for over 50% of all deaths [4].Several modifiable factors have been linked to altering inflammatory status including smoking, alcohol consumption, physical activity, and use of some medications [5]. Dietary compounds are also one of the important factors influencing systemic inflammation. For instance, Western-style diets are usually associated with higher inflammatory status, while Mediterranean diets are related to lower levels of inflammation [6]. Whey protein, as a group of globular proteins, is considered one of the major proteins in milk [7]. It is composed of many proteins or peptides including β-lactoglobulins, α-lactoalbumins, glycomacropeptide, immunoglobulins, serum albumins, and lactoferrin with several functions in the body [8][9]. Numerous potential biological activities have also been attributed to the consumption of this milk-derived substance including cardiovascular protection, antioxidative effects, immune-enhancing properties as well as weight loss effects [10][11]. 

Moreover, the anti-inflammatory effects of whey protein have been reported in several previous studies including those carried out in dialysis patients [12] or obese women with metabolic syndrome [13]. Cheese whey protein, as a source of threonine and cysteine, has also reduced gut inflammation in rats by promoting mucin synthesis and changing intestinal microflora [14]. previous meta-analysis, performed by Zhou *et al*. [15], has evaluated the chronic effects (≥4 weeks) of whey protein supplementation on a single inflammatory marker (i.e. C-reactive protein (CRP)). Results from nine included randomized trials in this study indicated that whey protein significantly reduced CRP levels in those with high doses of intervention or high baseline CRP concentrations.In the past few years, however, there has been an increasing trend to investigate the acute or short-term effects of whey protein on inflammatory status, especially in athletes [16][17][18][19][20][21]. Many of these interventions were also combined with carbohydrate [16][17][18][19], as a source of energy [22]. It has been reported that co-ingestion of protein and carbohydrates following exercise may increase muscle glycogen synthesis and functional capacity [23]. However, the research result obtained in this area is contradictory. Several clinical trials indicated that supplementing whey protein alone or along with carbohydrates can reduce inflammatory markers for a short duration [24][25][26][27], while some others could not show any beneficial effects [16][28][29][30]. Accordingly, this review, for the first time, aimed to systematically evaluate the acute or short-term effects of whey protein supplementation alone or in combination with carbohydrates on inflammatory markers including interleukin-6 (IL-6), tumor necrosis factor-alpha (TNF-α) and CRP.

## Materials and Methods

This systematic review was developed according to the Preferred Reporting Items for Systematic Reviews and Meta-analyses (PRISMA) statement [31], and was registered in the database of the International Prospective Register of Systematic Reviews (PROSPERO) with the registration number CRD42021273915 (available at: https://www.crd.york.ac.uk/prospero). The research protocol has not been yet published.

*Search Strategy and Study Selection*

The search strategy was developed based on the medical subject heading (MeSH) database and search terms of relevant review studies. The full search strategy is included in Supplementary file 1, which consists of two components: i.e. the intervention (whey protein) and the outcome (inflammatory markers). The systematic literature search was performed in PubMed, Scopus, and Web of Science databases from 1 January 1990 up to 23 September 2021 without language restriction. Reference lists of related review articles were also searched for possible additional relevant studies [15][32][33][34].

Studies meeting all the following criteria were included in this systematic review: (1) clinical trials with either parallel or cross-over design; (2) healthy or unhealthy adult participants (mean age>18 years); (3) oral administration of whey protein in any form (whey protein concentrate, isolate, hydrolysate, etc.) (WP group) or whey protein combined with carbohydrate (WP+CHO group); (4) included at least one comparison group; (5) assessed serum inflammatory markers including IL-6, TNF-α, CRP or high-sensitivity CRP (hs-CRP); and (6) a maximum duration of 15 days. Conference papers without available full texts were excluded from the present review.

Two reviewers screened the titles and abstracts of retrieved studies according to the eligibility criteria. Subsequently, full-texts of those studies without sufficient information based on title and abstract were assessed by two independent reviewers (MM and SMA). Discrepancies were clarified through discussion and when clarification was not possible, a third reviewer (AK) was consulted.

*Data Extraction*

Data extraction was performed by one reviewer (MM) and checked for accuracy by a second reviewer (MRZ). Disagreements between the reviewers were eventually resolved by discussion. The following data were extracted from the included studies:first author, year of publication, country (where the study was done), study design, participants’ characteristics, sample size, age, sex, body mass index (BMI), intervention details including dose and type of whey protein or carbohydrate consumed (and concurrent exercise program, if present), information on comparison group, intervention duration, time points of inflammatory marker assessments and the findings related to comparing the inflammatory markers between the groups.

*Quality Assessment*

The quality (risk of bias) of all included study was assessed by the Cochrane risk of bias tool for randomized controlled trials [35]. The tool comprises seven domains including randomized sequence generation, allocation concealment, blinding of participants and personnel, blinding of outcome assessor, incomplete outcome data, selective outcome reporting, and other sources of bias.

Subsequently, the studies were classified into three groups of qualities (good, fair, and poor). The study’s risk of bias was assessed independently by two reviewers (MM and NS). Any disagreements were resolved by consensus, and if necessary a third reviewer (AK) arbitrated.

## Results

*Selection Process*

**Figure-1:**
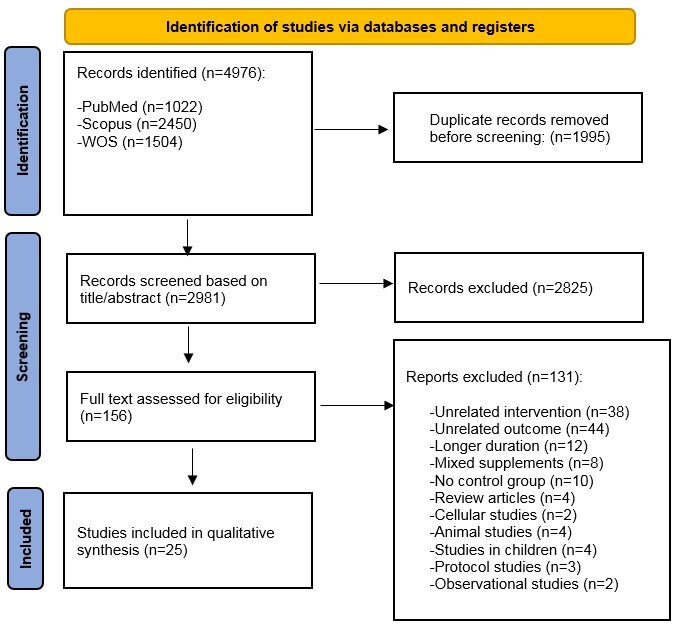
Figure 1. Flow diagram of the literature search and selection of studies

A total of 4976 articles were retrieved from electronic databases. After removing duplicates, 2981 studies were screened by titles or abstracts. The full texts of 156 potentially relevant articles were then checked for eligibility. Ultimately, 25 clinical trials met the inclusion and exclusion criteria for inclusion in this systematic review. The selection procedure of the studies is presented in Figure-1. 

*Study Characteristics*

The study characteristics of the included studies are summarized in Table-1 and Table-2. Of the 25 studies included, 15 administered whey protein alone and 10 provided whey protein plus carbohydrates. These studies were published between 2009 and 2021 and were performed in different geographical locations: seven in Europe, seven in Asia, five in North America, three in South America, and three in Australia. 

Regarding participant characteristics, about half of the studies were conducted in athletes or active adults (12 studies (48%); five in WP and seven in WP+CHO group), seven studies were carried out on different ill patients and three studies were conducted in patients with obesity or overweight ones. The remaining three studies also studied sedentary men, healthy males, or medical students.

The mean ages of participants ranged from 21.44-81.95 yrs. (22.3-76.2 yrs. in WP and 21.44-81.95 yrs. in WP+CHO group). More than half of the studies (13 studies (52%); seven in WP and six in WP+CHO group) were conducted exclusively in males, nine studies were carried out on both genders and the remaining three studies were only performed on females. Among those studies assessed individuals’ BMI (ten studies), the mean of BMIs was 20-35.61 kg/m2.

Considering the type of whey protein consumed, whey protein isolate (WPI) was the most common type of protein used in the studies (ten studies (40%); six in WP and four in the WP+CHO group). Whey protein hydrolysate (WPH), whey protein concentrate (WPC), hydrolysate of WPI, yellow fermented whey protein, and a blend of WPI and WPC were other forms of reported protein. Nonetheless, seven studies did not declare the type of whey protein that was consumed. 

Regarding intervention doses, a wide range of whey protein or carbohydrate doses (CHO as the intervention group) was used. Some studies had a fixed dose for all the participants (7-114 g/day for WP and 25-250 g/day for CHO), and the others administered the interventions based on the individuals’ body weight (0.6-2.3 g/kg/day for WP and 1-7 g/kg/day for CHO). 

Each study had at least one comparison group. Participants in these groups received either protein-containing supplements (such as casein, α-lactalbumin, or white bread and sour milk cheese) or non-protein interventions (such as maltodextrin, water, or having no supplementation). Over half of the studies (56%, 14 studies) reported a concurrent exercise program in parallel with the supplement protocol. This was even more pronounced in the WP+CHO group where 70% (seven out of ten) of the studies had a supplemental exercise program, whereas 46.67% of the studies in the WP group (seven out of 15) had an exercise schedule.

The duration of the studies varied between one to 15 days. One day acute intervention were predominantly (90%, nine out of ten studies) in the WP+CHO group, whereas nearly half of the studies in the WP group (46.67%, seven out of 15 studies) had an acute duration. 

Besides, in more than half of the studies (13 studies (52%); seven in WP and six in the WP+CHO group) the inflammatory markers were measured at multiple time points (>2 times), while in ten studies (seven in WP and three in WP+CHO group) the markers were assessed two times (before and after the supplementation period). 

Furthermore, in one study conducted by de Carvalho *et al*. [30], the inflammatory status was evaluated only at the end of the intervention, and in another one [38] although baseline values were checked out but not reported in the article.

**Table:** Table 1. Characteristics of included studies evaluating the acute or short-term effects of whey protein on inflammatory markers

**First author,** **Year, Ref**	**Country/ Study design**	**Participants’** **characteristic/** **sample size^§^**	**Age, Sex, BMI**	**Intervention group(s) ** **(type and dose)**	**Comparison group(s)** **(type and dose)**	**Duration/ Time points of inflammatory marker assessments**	**Outcome assessed / Result **	**Overall Quality**
Mizubuti, 2021 [28]	Brazil/ Parallel	Patients with chronic liver disease on the waiting list for liver transplantation/n=75	52.13 M/F NR	WPI (20 g twice a day)	Casein (20 g twice a day)	15 days/Before and after supplementation period	IL-6, TNF-α/No significant differences were observed between the groups.	Fair	
Hilkens, 2021 [29]	Netherlands/ Parallel	Recreationally active, non-obese young men/n=39	23.51 M 22.84	WPC received in mid-morning (25 g/d) and ~1h before sleep (50 g/d) for 9 days. On day 5 participants performed a bout of eccentric exercise (100 drop jumps).	Maltodextrin received in mid-morning and ~1h before sleep (totally 72 g/d) for 9 days. On day 5 participants performed a bout of eccentric exercise (100 drop jumps).	9 days/On day 5 at pre-exercise, and 0, 1, 24, 48 and 72h post-exercise	CRP/No significant treatment×time interaction effect was observed.	Poor	
Nieman, 2020 [36]	USA/ Parallel	Non-athletic, non-obese, healthy men/n=92	38.57 M 25.4	Participants engaged in two physical fitness tests and one 90-min eccentric exercise bout every day and also supplemented with WPI (0.3 g/kg) three times a day.	a) Placebo (237 mL water) three times a day. b) Pea protein isolate (0.3 g/kg) three times a day. Both groups engaged in the same exercise protocol as the intervention group.	5 days/All the 5 days in fasting state and also in day 1 following the 90-min eccentric exercise	CRP/No significant treatment effect or treatment×time interaction effect was observed.	Poor	
Saracino, 2020 [37]	USA/ Parallel	Middle-aged, recreationally active healthy men/n=27	55 M NR	a)WPH b)WPI Participants performed maximal voluntary eccentric contractions of the knee extensors and flexors. WPH or WPI were provided after exercise (25 g) and also before sleep for 3 days (40 g/night).	a) A flavor matched, non-caloric placebo b) Plant-based protein (rice and pea combination) Both supplements were provided after exercise (25 g) and also before sleep for 3 days (40 g/night). Exercise protocols were the same as the intervention groups.	3 days/Pre-exercise and at 0, 4, 6, 24, 48, and 72h post-exercise	IL-6/No significant group×time interaction or main effect of group was observed.	Poor	
Celik, 2019 [38]	Turkey/ Parallel	Third grade medical students/n=36	NR M/F NR	Whey protein (44 g/d)	a) No nutritional supplementation b) Casein (33 g/d)	15 days/At baseline and on the examination day (day 16). However, the baseline values were not reported.	IL-6, TNF-α/TNF-α in the whey group was significantly higher than the group consuming nothing. No significant change in IL-6 was observed between the groups.	Poor	
Snipe, 2017 [21]	Australia/ Crossover	Non-heat adopted endurance-trained runners/n=9	31 M/F NR	WPH consumed pre-exercise (15 g) and every 20 min (15 g) during 2h running at 35.5°C.	a) Glucose b) Water They were consumed pre-exercise and every 20 min during 2h running at 35.5°C (15 g for glucose; ad lib for water at each time)	1 day/Before and immediately after exercise	IL-6, TNF-α/Whey protein group was not significantly different compared to other groups.	Poor	
Mariotti, 2015 [39]	France/ Crossover	Healthy overweight young men with waist circumference >94 cm/n=10	34 M 30.2	Whey protein (55 g; consumed in a high fat meal)	a) Casein (54 g; consumed in a high fat meal) b) α-lactalbumin-enriched whey protein (49 g; consumed in a high fat meal)	1 day/Before the meal and at 2, 4 and 6h after the meal	IL-6, TNF-α/No significant meal effects or meal×time interaction effects were found.	Poor	
Schroer, 2014 [20]	USA/ Crossover	Endurance-trained cyclists/n=8	22.3 M/F NR	Participants had 120 min of constant-load cycling and a 30-km time trial. WPH was received before exercise and every 15 min during the 120 min cycling; Total WPH received was 101.25 g.^*^	a) Non-caloric artificially sweetened solution (250 ml each time) b) L-alanine (Totally received 33.75 g) The exercise protocols and time points of supplementations were similar to the intervention group.	1 day/Before and after constant-load cycling	IL-6/The fold-change was attenuated (92% likelihood) with whey protein compared to non-caloric solution.	Poor	
Kinsey, 2014 [40]	USA/ Parallel	Sedentary overweight or obese healthy women/n=42	28.71 F 35.61	Night-time intake of whey protein (30 g whey protein as the main ingredient- 50% blend of WPI and WPC)	a) Night-time intake of maltodextrin (30 g maltodextrin as the main ingredient) b) Night-time intake of casein (30 g micellar casein as the main ingredient)	1 day/Baseline and also in the morning after night-time ingestion of the supplement	hs-CRP/No significant differences were observed between the groups.	Poor	
Baba, 2014 [41]	Japan/ Crossover	Non-athletic adults, but capable of running for at least 1 h at 10 km/h and able to maintain an intensity of at least 70% of VO_2max _/n=14	31 M 22.8	Once before, 3 times during, and once after a 60-min running session, a powder consumed that contained 22.8 g of WPI as the main ingredient.	Once before, 3 times during, and once after a 60-min running session, a placebo consumed that contained 5.2 g of the same powder in the intervention group, after removal of whey protein.	1 day/30 min before, immediately after, and at 0.5, 2, 4, 6, and 24h after the completion of running	IL-6/No significant differences were observed between the groups.	Poor	
Singh, 2014 [42]	India/ Parallel	Severe acute pancreatitis patients/n=68	38.27 M/F NR	Whey protein (10 g twice a day)	Glutamine (10 g twice a day)	7 days/Before and after supplementation period	hs-CRP/No significant differences were observed between the groups.	Good	
Pal, 2011 [43]	Australia/ Crossover	Healthy overweight or obese postmenopausal women/n=20	57.5 F 32.5	WPI (45 g; consumed with a breakfast meal).	a) Glucose (45 g; consumed with a breakfast meal). b) Sodium caseinate (45 g; consumed with a breakfast meal).	1 day/Before and at 1, 2, 3, 4 and 6h after supplementation	IL-6, TNF-α, CRP/No significant group effects were observed.	Poor	
de Aguilar-Nascimento, 2011 [24]	Brazil/ Parallel	Elderly patients admitted to ICU due to acute ischemic stroke/n=25	76.2 M/F NR	Early nasogastric feeding with standard formula containing WPH (1.2 g of protein/kg/d)	Early nasogastric feeding with standard formula containing hydrolyzed casein (1.2 g of protein/kg/d)	5 days/Before and after supplementation period	IL-6, CRP/IL-6 was significantly decreased in the whey group than the casein group. No differences were observed for CRP.	Fair	
Kullisaar, 2011 [25]	Estonia/ Parallel	Patients with light to moderate lower urinary tract symptoms/benign prostatic hypertrophy/n=51	50-60**^¶^** M NR	Yellow fermented whey product (50 g/d)	Apple juice (50 g/d)	2 weeks/Before and after supplementation period	hs-CRP/hs-CRP significantly decreased after consumption of whey in comparison with apple juice.	Poor	
Buckley, 2010 [44]	Australia/ Parallel	Sedentary men/n=28	18-30**^¶^** M NR	a)WPI b)WPI_HD_ 100 maximal eccentric contractions of the knee extensors were performed. Soon after, 6h, and 22h after the contractions, 25 g of WPI or WPI_HD_ were consumed.	100 maximal eccentric contractions of the knee extensors were performed. Soon after, 6h, and 22h after contractions, a flavored water was consumed.	1 day/Prior to maximal eccentric contractions, immediately after it, and at 1, 2, 6 and 24h post-eccentric contractions	TNF-α/No significant treatment×time interaction effect was observed.	Poor	

**§** Since in some studies the inflammatory markers were not assessed for all the included participants, the sample size of the evaluated inflammatory markers were considered. * WPH, non-caloric solution and L-alanine were also given at three points during the time trial but without blood sampling. All the 3 products contained sodium chloride, potassium chloride, and flavoring. **¶ **In cases where the mean age of the participants was not reported, the inclusion criteria for age of the participants were specified. **ad lib:** ad libitum; **BMI:** body mass index; **CRP:** C-reactive protein; **F:** female; **hs-CRP:** high-sensitivity C-reactive protein; **ICU:** intensive care unit; **IL-6:** interleukin-6; **M:** male; **NR:** not reported; **Ref:** reference; **TNF-α:** tumor necrosis factor-alpha; **WPC:** whey protein concentrate; **WPH:** whey protein hydrolysate; **WPI:** whey protein isolate; **WPIHD:** hydrolysate of whey protein isolate

**Table:** Table 2. Characteristics of included studies evaluating the acute or short-term effects of whey protein in combination with carbohydrate on inflammatory markers

**First author,** **Year, Ref**	**Country/ Study design**	**Participants’** **characteristic/** **Total sample size^§^**	**Age, Sex, BMI**	**Intervention group(s) (type and dose)**	**Comparison group(s) (type and dose)**	**Duration/ Time points of inflammatory marker assessments**	**Outcome assessed / Result **	**Overall Quality**
de Carvalho, 2021 [30]	Brazil/ Parallel	Patients ≥20 years with head and neck cancer and candidates for elective surgery/n=49	59.84 M/F 25.44	Clear fluid containing whey protein (7 g) and maltodextrin (25 g) received 4h before surgery.	Clear fluid containing maltodextrin (25 g) received 4h before surgery.	1 day/On the second post-surgery day	CRP/No significant differences were observed between the groups.	Poor
Deng, 2020 [26]	China/ Parallel	Aged patients undergoing elective hip fracture surgery/n=35	81.95 M/F 20	400 mL of WPH (14%) with glucose solution (10%) 24h before surgery, and 200 mL of the same solution 3h before surgery were received.	Distilled water (400 mL 24h before surgery, and 200 mL 3h before surgery)	1 day/On the day before surgery and 24h after surgery	CRP/After surgery, CRP was significantly lower in the intervention group than the control group.	Poor
Yi, 2020 [27]	Malaysia/ Parallel	Ambulated patients scheduled for elective surgery for suspected gynecologic cancer/n=118	50.31 F 26.57	12 and 3h before, and 4h after operation, a formulated drink with whey protein (totally 45 g) and CHO (totally 250 g) received. When at least 500 mL of the formulated clear fluid with another additional clear fluid was tolerated, solid food was allowed.	Dinner was consumed 12h before operation. After operation and presence of bowel sounds, patients were allowed to receive clear fluid, nourishing fluid, soft diet, and a regular solid diet according to tolerance.	1 day/Baseline and after operation	CRP/The intervention significantly prevented the post-operational increase in CRP compared with the control.	Fair
Isenmann, 2019 [45]	Germany/ Crossover	Healthy physically active sports students (amateur sportsmen)/n=27	23.2 M NR	After testing leg strength (by back squat) and ingesting a breakfast, participants performed a 10 km run. Then, whey protein (52 g) plus glucose (45 g) was ingested.	a) No supplementation b) White bread and sour milk cheese The protocols were the same as the intervention group except for the type of supplement.	1 day/Baseline and 3h after supplementation	IL-6/ IL-6 levels were significantly lower in "whey protein plus glucose" and "white bread and sour milk cheese" groups compared with "no supplementation" group.	Poor
Qin, 2019 [16]	Hong Kong/ Crossover	Healthy endurance runners/n=11	31 M NR	Participants ingested WPI (0.34 g/kg/h) and CHO (0.66 g/kg/h) within 2h. Then they ran 21 km on a treadmill.	Participants ingested α-lactalbumin (0.34 g/kg/h) and CHO (0.66 g/kg/h) within 2h. Then they ran 21 km on a treadmill.	1 day/2h before exercise, immediately before exercise, immediately post-exercise, and 24h post-exercise	IL-6/No significant treatment effect or treatment×time interaction effect was observed.	Poor
Qin, 2017 [17]	Hong Kong/ Crossover	Healthy endurance runners/n=12	30.4 M NR	Participants ran for 90 min. During the first 2h of recovery, WPI (0.34 g/kg/h) and CHO (0.66 g/kg/h) were consumed.	Participants ran for 90 min. During the first 2h of recovery, α-lactalbumin (0.34 g/kg/h) and CHO (0.66 g/kg/h) were consumed.	1 day/Pre- and post-exercise, every 2h during the 4h recovery, and 24h post-exercise	IL-6/No significant differences were observed between the groups.	Poor
Dahlquist, 2017 [18]	Canada/ Crossover	Highly trained cyclists/n=10	26.9 M NR	Participants performed a cycling test that had warm-up and cooling-down periods. Then they consumed WPI (25 g) and CHO (75 g).	a) non-nitrogenous, zero calorie drink (550 ml) b) WPI (25g), CHO (75g), vitamin D_3_ (5000IU) and vitamin K_2 _(1000µg). The exercise protocols were the same as the intervention group.	1 day/Baseline, immediately post-exercise and 3h post-exercise	IL-6/No significant differences were observed between the groups.	Poor
Hansen, 2015 [19]	Denmark and Portugal / Parallel	Elite orienteering runners/n=10 for IL-6;n=5 for TNF-α	21.44 M/F NR	Thirteen exercise sessions were performed during 1-week training camp. A 4-km run-test was performed before and on the last day. Before and after each session, WPH (0.3 g/kg) and WPH+CHO (0.3 and 1 g/kg) were provided, respectively.	The exercise protocol was the same as the intervention group. Before and after each exercise session, 0.3 and 1.3 g/kg CHO were provided, respectively.	7 days/Baseline pre test, baseline post test, day 1 and day 7 in the morning and day 7 post-test	IL-6, TNF-α/No significant treatment×time interaction effect was observed. The levels of cytokines were not affected by the treatments.	Poor
Kerasioti, 2013 [46]	Greece/ Crossover	Physically active men/n=9	28 M 23	Participants underwent 2h cycling, 4h recovery, 1h cycling, cycling until exhaustion (time Trial) and 1h recovery. During the 4h recovery a cake with 0.26 g whey protein/kg/h and 0.9 g CHO/kg/h was consumed.	The exercise protocol was the same as the intervention group. During the 4h recovery a cake with 1.1 g CHO/kg/h and 0.1 g protein/kg/h was consumed.	1 day/Pre-exercise, 30 min post-exercise, 4h post-exercise, immediately post-time Trial and 48h post-time Trial	IL-6, CRP/The experimental cake significantly reduced IL-6 and CRP at 4 h post-exercise than the placebo cake.	Poor
Betts, 2009 [47]	UK/ Crossover	Highly trained healthy young athletes (cyclists or team-sport players) /n=17	26 M NR	Participants underwent a 20 min of warm-up period, 90 min of shuttle-running, and a 4h recovery period. During all phases they ingested 0.4 g/kg/h WPI plus 1.2 g/kg/h CHO.	Participants underwent a 20 min of warm-up period, 90 min of shuttle-running, and a 4h recovery period. During all phases they ingested 1.2 g/kg/h CHO.	1 day/CRP was measured pre and post exercise, after 4h recovery and 24h post exercise. IL-6 was measured pre and post exercise, 4 times during recovery and 24h post exercise.	IL-6, CRP/No significant differences were observed between the groups.	Poor

**§** Since in some studies the inflammatory markers were not assessed for all the included participants, the sample size of the evaluated inflammatory markers were considered. **BMI:** body mass index; **CHO: **carbohydrate; **CRP:**C-reactive protein; **F:** female; **IL-6:** interleukin-6; **M:** male; **NR:** not reported; **Ref:** reference; **TNF-α:** tumor necrosis factor-alpha; **WPH:** whey protein hydrolysate; **WPI:** whey protein isolate

*Effects of Interventions on Inflammation*

In the current review, seven out of 25 studies (28%) observed a significant reduction in at least one inflammatory marker (IL-6 in four studies, CRP in three studies and hs-CRP in one study) following whey protein consumption or combination of it with carbohydrate. On the other hand, one study (4%) reported a significant increase in inflammatory status (TNF-α levels) after supplementing with whey protein. 

It is worth mentioning that three out of seven studies (42.86%) with significant protective effects, administered whey protein in the form of hydrolysate (Table-1 and -2).

Of the studies comparing the interventions (WP or WP+CHO) with non-protein controls (17 trials), four studies (23.53%) showed significant improvements in the inflammatory markers. It should be noted that the one study carried out by Celik et al. [38] that observed deterioration in inflammatory status (one out of 17) belonged to the non-protein comparison group.

Furthermore, among those trials comparing the interventions (WP or WP+CHO) with protein-containing groups (15 studies), three studies (20%) reported a significant decrease in inflammatory biomarkers. One of the comparison groups in this category (one out of 15 studies) included participants who consumed dinner, clear fluid, nourishing fluid, a soft diet and regular solids during the intervention period [27]. We categorized this group as a protein-containing comparison group since dinners and regular solid diets usually contain some protein.

Among those studies compared the effects of whey protein alone with non-protein or protein-containing comparison groups, 18.18% (two out of 11) and 10% (one out of ten) of the trials revealed a significant reduction in the inflammatory markers, respectively. The only study that reported elevated TNF-α levels (one out of 11), comparing the effects of whey protein alone with a control group, in which the the control group was given no nutritional supplementation. 

Moreover, of those studies comparing whey protein and carbohydrate with non-protein or protein-containing comparison groups, 33.33 % (two out of six) and 40% (two out of five) of the articles, a significant decrease in the inflammatory response was observed.

*The Overall Quality of Studies*

Using the Cochrane risk of bias tool, most studies (84%) were rated as ‘poor’ (21 studies; 12 in the WP group, nine in the WP+CHO group); three studies were classified as ‘fair’ (two in the WP group, one in the WP+CHO group) and one study (in the WP group) was rated as ‘good’. Supplementary file 2 provides quality assessment details of mentioned studies.

## Discussion 

The present systematic review indicated that whey protein alone or in combination with carbohydrates may not affect the inflammatory response after a short time. Less than one-third (28%) of the included studies reported significant reductions in inflammatory markers. Moreover, less than one-fourth of the trials comparing the interventions with non-protein controls or protein-containing comparison groups observed significant improvements in inflammatory status (23.53% and 20%, respectively). 

The most remarkable finding was related to the studies comparing the effects of whey protein plus carbohydrate with protein-containing comparison groups, in which 40% of the articles (two out of five) indicated a significant decrease in the inflammatory markers. However, the percentage is not high enough and this subgroup of studies included very few trials (only five) to make a definite conclusion. 

In a meta-analysis carried out in 2015 [15] results of nine included studies with a duration of at least four weeks demonstrated that whey protein and its derivate significantly decreased CRP levels in participants supplemented with ≥20 g/day or in those with baseline CRP concentrations ≥3 mg/L. The supplementation also showed a small but nonsignificant effect on reducing CRP considering all the nine included studies. 

Another meta-analysis in 2021 [48] evaluated the effects of chronic ingestion (>4 weeks) of whey protein on TNF-α and IL-6 status. Acute and communicable conditions as well as non-English language articles were excluded from this review. Results of the eleven included studies showed no significant effect of this protein on the markers compared to carbohydrate or protein comparison groups. Moreover, a narrative review in older individuals by Ticinesi, *et al*. in 2016 [34] investigated the effects of whey protein intake on inflammatory markers and included four randomized controlled trials. But the findings did not support an anti-inflammatory effect for this protein in aged participants, and no definite conclusion was made. 

These results are in agreement with the findings of the present review. Several review studies have also been conducted on dairy protein (such as a combination of casein and whey protein) intakes. Nevertheless, these reviews did not interpret their results for each type of protein alone. In a systematic review by Nieman *et al*. [33], the impact of dairy protein was assessed on inflammatory biomarkers in those without severe inflammatory-related diseases. The results of eight included studies showed neutral effects on the biomarkers similar to our findings. Besides, a non-systematic literature review [49] evaluated the effects of ingesting milk proteins on the management of cardiometabolic diseases. Of the nine trials investigating the effects of dairy protein (including casein, whey, whey-derived peptide, ribonuclease-enriched lactoferrin, etc.) on inflammatory status, no decisive and clear conclusion was obtained for the efficacy of these proteins.

Oxidative stress and inflammatory processes are closely related. Reactive oxygen or reactive nitrogen species can increase pro-inflammatory gene expression by affecting intracellular signaling pathways [50]. The antioxidant activity of whey protein is revealed by cysteine residues, which are essential for glutathione synthesis [51][52]. Consequently, this dairy protein may also be involved in reducing the inflammatory response. Moreover, immunomodulatory properties have been attributed to lactoferrin and immunoglobulins, which are isolated from whey. These immune proteins have been reported to modulate inflammatory processes by reducing intestinal permeability or affecting gene expression [53]. Whey protein is also a rich source of branched-chain amino acids (BCAAs), especially leucine and isoleucine [54]. Depending on blood levels, Leucine can stimulate both anti-inflammatory and pro-inflammatory processes [55]. 

It has been suggested that acute ingestion of β-hydroxy β-methyl butyrate, a metabolite of leucine breakdown, before and after resistance training may reduce the pro-inflammatory response [56]. 

On the other hand, an in-vitro study performed on cultured peripheral blood mononuclear cells showed that BCAAs induced the activation of nuclear factor-κB, causing the release of pro-inflammatory cytokines such as IL-6 and TNF-α [57]. 

Therefore, these contradictory proposed mechanisms may be one of the contributing factors explaining the ineffectiveness of whey protein on inflammatory responses. Additionally, it should be noted that there are some methodological inconsistencies among the studies included in this review that may affect the results. 

For instance, types and doses of whey protein consumed, types of comparison groups (either non-protein or protein-containing groups), participants’ characteristics (including health status, age, sex, and BMI), sample sizes and presence of concurrent exercise programs were different among the articles. Furthermore, the neutral conclusions may have been due to the poor quality of most studies or the insufficient duration of the study to observe the significant results. Therefore, researchers are encouraged to focus on conducting high-quality studies with longer durations in the future.

In this review, almost half of the studies showing significant protective effects had administered WPH in the form of hydrolysate (WPH). It has been reported that protein hydrolysates can have several biological characteristics including antimicrobial, anti-thrombotic or antioxidative effects [58]. Besides, in line with the findings of our study, results of in vitro research indicated that whey protein hydrolysate can downregulate TNFα-induced inflammation by altering gene expression [59]. 

It should be noted that ten trials of this review provided whey protein along with carbohydrate, and 70% of them had a concurrent exercise program. It has been demonstrated that carbohydrates consumption replenishes glycogen stores and can enhance endurance performance in athletes [60][61]. When protein is also ingested with carbohydrates, insulin secretion and muscle glycogen synthase activity can be increased, which may lead to speeding up muscle glycogen resynthesis following exercise [62]. 

In the present study, no quantitative analysis was performed on the data. Some of the included studies did not have adequate criteria for conducting a meta-analysis. For example, in some articles the exact values for inflammatory markers or measurement of variability were not clear on the graphs [37][43][46]; baseline values were not assessed or reported [30][38]; or the measurement unit for variability was not specified [25]. 

The high diversity of the comparison groups was another reason for not performing a meta-analysis. Furthermore, more than half of the included studies (52%) measured the inflammatory markers at multiple time points during the intervention. As a result, conducting a systematic review could have provided comprehensive insight to compare all time points.

To the best of our knowledge, this is the first systematic review that analyzed the acute or short-term effects of whey protein supplementation alone or in combination with carbohydrates on inflammatory biomarkers. Besides, no restriction was placed on the language of retrieved studies or the health status of the participants, which could increase the generalizability of the findings. However, this review had some limitations. 

First of all, the majority of the included studies had low quality. Thus the obtained results should be interpreted with caution. Secondly, some other mediators except those common ones assessed in this study can be categorized as inflammatory (such as IL-1β or serum amyloid A [1]). Finally, this review included studies performed only on adult participants and trials performed on children were excluded.

## Conclusion

In conclusion, it seems that consumption of whey protein alone or in the combination with carbohydrates for a short duration may not alter the inflammatory status. However, conducting further primary studies with high quality as well as performing quantitative analysis in this area (if possible in the future) is required to make a definite conclusion.

## Conflict of Interest 

The authors declare that they have no competing interests.

## Supplementary Materials

Supplementary file 1 Search strategies

Supplementary file 2 Quality assessments of studies

## References

[R1] Abdulkhaleq LA, Assi MA, Abdullah R, Zamri-Saad M, Taufiq-Yap YH, Hezmee MNM (2018). The crucial roles of inflammatory mediators in inflammation: A review. Vet World.

[R2] Oke SL, Tracey KJ (2009). The inflammatory reflex and the role of complementary and alternative medical therapies. Ann N Y Acad Sci.

[R3] Zhang K, Kaufman RJ (2008). From endoplasmic-reticulum stress to the inflammatory response. Nature.

[R4] Furman D, Campisi J, Verdin E, Carrera-Bastos P, Targ S, Franceschi C, et al (2019). Chronic inflammation in the etiology of disease across the life span. Nat Med.

[R5] Kantor ED, Lampe JW, Kratz M (2013). Lifestyle factors and inflammation associations by body mass index. PLoS One.

[R6] Wirth MD, Hébert JR, Shivappa N, Hand GA, Hurley TG, Drenowatz C, et al (2016). Anti-inflammatory Dietary Inflammatory Index scores are associated with healthier scores on other dietary indices. Nutr Res.

[R7] Davoodi SH, Shahbazi R, Esmaeili S, Sohrabvandi S, Mortazavian A, Jazayeri S, et al (2016). Health-related aspects of milk proteins. Iran J Pharm Res.

[R8] Patel SJ (2015). Functional food relevance of whey protein: A review of recent findings and scopes ahead. J Funct Foods.

[R9] León-López A, Pérez-Marroquín XA, Campos-Lozada G, Campos-Montiel RG, Aguirre-Álvarez GJF (2020). Characterization of whey-based fermented beverages supplemented with hydrolyzed collagen: Antioxidant activity and bioavailability. Foods.

[R10] Wirunsawanya K, Upala S, Jaruvongvanich V, Sanguankeo A (2018). Whey protein supplementation improves body composition and cardiovascular risk factors in overweight and obese patients: A systematic review and meta-analysis. J Am Coll Nutr.

[R11] Zhang J, Tong X, Wan Z, Wang Y, Qin L, Szeto IMY (2016). Effect of whey protein on blood lipid profiles: a meta-analysis of randomized controlled trials. Eur J Clin Nutr.

[R12] Hassan K (2017). Does whey protein supplementation improve the nutritional status in hypoalbuminemic peritoneal dialysis patients. Ther Apher Dial.

[R13] Aghamohammadi V, Haidari F, Mohammadshahi M, Ahmadi-Angali K, Asghari-Jafarabadi M (2018). Whey supplementation combined with energy-restricted diet alleviates 2-arachidonoylglycerol, adipocytokines, inflammatory factors and body composition in obese women with metabolic syndrome: a randomized controlled trial. Endocrinol Metab Syndr.

[R14] Sprong R, Schonewille A, Van der (2010). Dietary cheese whey protein protects rats against mild dextran sulfate sodium–induced colitis: Role of mucin and microbiota. J Dairy Sci.

[R15] Zhou L-M, Xu J-Y, Rao C-P, Han S, Wan Z, Qin L-Q (2015). Effect of whey supplementation on circulating C-reactive protein: A meta-analysis of randomized controlled trials. Nutrients.

[R16] Qin L, Sun F-H, Huang Y, Sheridan S, Sit CH-P, Wong SH-S (2019). Effect of pre-exercise ingestion of α-lactalbumin on subsequent endurance exercise performance and mood states. Br J Nutr.

[R17] Qin L, Wong SHS, Sun F-H, Huang Y, Sheridan S, Sit CHP (2017). Effects of alpha-lactalbumin or whey protein isolate on muscle damage, muscle pain, and mood states following prolonged strenuous endurance exercise. Front Physiol.

[R18] Dahlquist DT, Stellingwerff T, Dieter BP, McKenzie DC, Koehle MS (2017). Effects of macro-and micronutrients on exercise-induced hepcidin response in highly trained endurance athletes. Appl Physiol Nutr Metab.

[R19] Hansen M, Bangsbo J, Jensen J, Bibby BM, Madsen K (2015). Effect of whey protein hydrolysate on performance and recovery of top-class orienteering runners. Int J Sport Nutr Exerc Metab.

[R20] Schroer AB, Saunders MJ, Baur DA, Womack CJ, Luden ND (2014). Cycling time trial performance may be impaired by whey protein and L-alanine intake during prolonged exercise. Int J Sport Nutr Exerc Metab.

[R21] Snipe RM, Khoo A, Kitic CM, Gibson PR, Costa RJ (2017). Carbohydrate and protein intake during exertional heat stress ameliorates intestinal epithelial injury and small intestine permeability. Appl Physiol Nutr Metab.

[R22] Livesey G (2021). Assessment of carbohydrate availability, fermentability, and food energy value in humans using measurements of breath hydrogen. J Am Coll Nutr.

[R23] Alghannam AF, Gonzalez JT, Betts JA (2018). Restoration of muscle glycogen and functional capacity: role of post-exercise carbohydrate and protein co-ingestion. Nutrients.

[R24] de Aguilar-Nascimento, Prado Silveira, Dock-Nascimento DB (2011). Early enteral nutrition with whey protein or casein in elderly patients with acute ischemic stroke: A double-blind randomized trial. Nutrition.

[R25] Kullisaar T, Punab M, Türk S, Veskioja A, Songisepp E, Zilmer M (2011). A fermented whey product benefits male patients with lower urinary tract symptoms. Food technologists, biotechnologists and nutritionists.

[R26] Deng Y, Fang Y, Li H, Chen J, An J, Qiao S, et al (2020). A preoperative whey protein and glucose drink before hip fracture surgery in the aged improves symptomatic and metabolic recovery. Asia Pac J Clin Nutr.

[R27] Yi HC, Ibrahim Z, Abu Zaid, Mat Daud, Md Yusop, Omar J, et al (2020). Impact of enhanced recovery after surgery with preoperative whey protein-infused carbohydrate loading and postoperative early oral feeding among surgical gynecologic cancer patients an open-labelled randomized controlled trial. Nutrients.

[R28] Mizubuti Y, Vieira E, Silva T, d’Alessandro M, Generoso S, Teixeira A, et al (2021). Comparing the effects of whey and casein supplementation on nutritional status and immune parameters in patients with chronic liver disease: a randomised double-blind controlled trial. Br J Nutr.

[R29] Hilkens L, De Bock, Kretzers J, Kardinaal AF, Floris-Vollenbroek EG, Scholtens PA, et al (2021). Whey protein supplementation does not accelerate recovery from a single bout of eccentric exercise. J Sports Sci.

[R30] de Carvalho, Silva TH, André JCS, de Barros, Ferreira AA, Murad LB, et al (2021). Preoperative fasting abbreviation with whey protein reduces the occurrence of postoperative complications in patients with head and neck cancer: a randomized clinical trial. Nutr Clin Pract.

[R31] Moher D, Liberati A, Tetzlaff J, Altman DG, PRISMA Group (2009). Preferred reporting items for systematic reviews and meta-analyses: the PRISMA statement. PLoS Med.

[R32] Moosavian SP, Rahimlou M, Saneei P, Esmaillzadeh A (2020). Effects of dairy products consumption on inflammatory biomarkers among adults: A systematic review and meta-analysis of randomized controlled trials. Nutr Metab Cardiovasc Dis.

[R33] Nieman KM, Anderson BD, Cifelli CJ (2021). The effects of dairy product and dairy protein intake on inflammation: A systematic review of the literature. J Am Coll Nutr.

[R34] Ticinesi A, Meschi T, Lauretani F, Felis G, Franchi F, Pedrolli C, et al (2016). Nutrition and inflammation in older individuals: focus on vitamin D, n-3 polyunsaturated fatty acids and whey proteins. Nutrients.

[R35] Higgins JP, Altman DG, Gøtzsche PC, Jüni P, Moher D, Oxman AD, et al (2011). The Cochrane Collaboration’s tool for assessing risk of bias in randomised trials. BMJ.

[R36] Nieman DC, Zwetsloot KA, Simonson AJ, Hoyle AT, Wang X, Nelson HK, et al (2020). Effects of whey and pea protein supplementation on post-eccentric exercise muscle damage: a randomized trial. Nutrients.

[R37] Saracino PG, Saylor HE, Hanna BR, Hickner RC, Kim J-S, Ormsbee MJ (2020). Effects of pre-sleep whey vs plant-based protein consumption on muscle recovery following damaging morning exercise. Nutrients.

[R38] Celik R, Kaymakci MS, Akalin D, Karademir E, Tuncer B, Bicim G, et al (2019). Effect of casein and whey proteins on examination stress. Marmara Med J.

[R39] Mariotti F, Valette M, Lopez C, Fouillet H, Famelart M-H, Mathé V, et al (2015). Casein compared with whey proteins affects the organization of dietary fat during digestion and attenuates the postprandial triglyceride response to a mixed high-fat meal in healthy, overweight men. J Nutr.

[R40] Kinsey AW, Eddy WR, Madzima TA, Panton LB, Arciero PJ, Kim J-S, et al (2014). Influence of night-time protein and carbohydrate intake on appetite and cardiometabolic risk in sedentary overweight and obese women. Br J Nutr.

[R41] Baba S, Ebihara S, Sakano K, Natsume M (2014). Whey protein-containing product reduces muscle damage induced by running in male adults. Sport Sci Health.

[R42] Singh N, Mishra SK, Sachdev V, Sharma H, Upadhyay AD, Arora I, et al (2014). Effect of oral glutamine supplementation on gut permeability and endotoxemia in patients with severe acute pancreatitis: a randomized controlled trial. Pancreas.

[R43] Pal S, Ellis V (2011). Acute effects of whey protein isolate on blood pressure, vascular function and inflammatory markers in overweight postmenopausal women. Br J Nutr.

[R44] Buckley JD, Thomson RL, Coates AM, Howe PR, DeNichilo MO, Rowney MK (2010). Supplementation with a whey protein hydrolysate enhances recovery of muscle force-generating capacity following eccentric exercise. J Sci Med Sport.

[R45] Isenmann E, Blume F, Bizjak D, Hundsdörfer V, Pagano S, Schibrowski S, et al (2019). Comparison of pro-regenerative effects of carbohydrates and protein administrated by shake and non-macro-nutrient matched food items on the skeletal muscle after acute endurance exercise. Nutrients.

[R46] Kerasioti E, Stagos D, Jamurtas A, Kiskini A, Koutedakis Y, Goutzourelas N, et al (2013). Anti-inflammatory effects of a special carbohydrate–whey protein cake after exhaustive cycling in humans. Food Chem Toxicol.

[R47] Betts JA, Toone RJ, Stokes KA, Thompson D (2009). Systemic indices of skeletal muscle damage and recovery of muscle function after exercise: effect of combined carbohydrate–protein ingestion. Appl Physiol Nutr Metab.

[R48] Jamshidi S, Mohsenpour MA, Masoumi SJ, Fatahi S, Nasimi N, Zahabi ES, et al (2021). Effect of whey protein consumption on IL-6 and TNF-α: A systematic review and meta-analysis of randomized controlled trials. Diabetes Metab Syndr.

[R49] Fekete ÁA, Givens DI, Lovegrove JA (2016). Can milk proteins be a useful tool in the management of cardiometabolic health An updated review of human intervention trials. Proc Nutr Soc.

[R50] Biswas SK (2016). Does the interdependence between oxidative stress and inflammation explain the antioxidant paradox. Oxid Med Cell Longev.

[R51] Veskoukis AS, Kerasioti E, Skaperda Z, Papapostolou PA, Nepka C, Spandidos DA, et al (2020). Whey protein boosts the antioxidant profile of rats by enhancing the activities of crucial antioxidant enzymes in a tissue-specific manner. Food Chem Toxicol.

[R52] Corrochano AR, Buckin V, Kelly PM, Giblin L (2018). Invited review: Whey proteins as antioxidants and promoters of cellular antioxidant pathways. J Dairy Sci.

[R53] Brimelow RE, West NP, Williams LT, Cripps AW, Cox AJ (2017). A role for whey-derived lactoferrin and immunoglobulins in the attenuation of obesity-related inflammation and disease. Crit Rev Food Sci Nutr.

[R54] Elovaris RA, Hajishafiee M, Ullrich SS, Fitzgerald PC, Lange K, Horowitz M, et al (2021). Intragastric administration of leucine and isoleucine does not reduce the glycaemic response to, or slow gastric emptying of, a carbohydrate-containing drink in type 2 diabetes. Diabetes Res Clin Pract.

[R55] Liberman K, Njemini R, Luiking Y, Forti LN, Verlaan S, Bauer JM, et al (2019). Thirteen weeks of supplementation of vitamin D and leucine-enriched whey protein nutritional supplement attenuates chronic low-grade inflammation in sarcopenic older adults: the PROVIDE study. Aging Clin Exp Res.

[R56] Arazi H, Taati B, Suzuki K (2018). A review of the effects of leucine metabolite (β-hydroxy-β-methylbutyrate) supplementation and resistance training on inflammatory markers: A new approach to oxidative stress and cardiovascular risk factors. Antioxidants (Basel).

[R57] Zhenyukh O, Civantos E, Ruiz-Ortega M, Sánchez MS, Vazquez C, Peiro C, et al (2017). High concentration of branched-chain amino acids promotes oxidative stress, inflammation and migration of human peripheral blood mononuclear cells via mTORC1 activation. Free Radic Biol Med.

[R58] Athira S, Mann B, Saini P, Sharma R, Kumar R, Singh AK (2015). Production and characterisation of whey protein hydrolysate having antioxidant activity from cheese whey. J Sci Food Agric.

[R59] Da Silva, Bigo C, Barbier O, Rudkowska I (2017). Whey protein hydrolysate and branched-chain amino acids downregulate inflammation-related genes in vascular endothelial cells. Nutr Res.

[R60] Kloby Nielsen, Tandrup Lambert, Jeppesen PB (2020). The effect of ingesting carbohydrate and proteins on athletic performance: a systematic review and meta-analysis of randomized controlled trials. Nutrients.

[R61] Lawler TP, Cialdella-Kam L (2020). Non-carbohydrate dietary factors and their influence on post-exercise glycogen storage: a review. Curr Nutr Rep.

[R62] McCartney D, Desbrow B, Irwin C (2018). Post-exercise ingestion of carbohydrate, protein and water: a systematic review and meta-analysis for effects on subsequent athletic performance. Sports Med.

