# Supplementary material for: Acute or Short-term Effects of Whey Protein Alone or Along with Carbohydrate on Inflammation: A Systematic Review of Clinical Trials: -
Source: Galen Med J. 2023 Apr 18;12:e2441. doi: 10.31661/gmj.v12i.2441 (PMC10506851; doi:10.31661/gmj.v12i.2441)
Supplement: Supplementary file 1 [file GMJ-12-e2441-s1.docx]

**Supplementary file 1**

**Search strategies:**

**PubMed**

(whey*[tiab] OR “whey protein*”[tiab] OR “milk protein*”[tiab] OR “dairy protein*”[tiab]) AND (inflam*[tiab] OR “pro inflam*”[tiab] OR pro-inflam*[tiab] OR proinflam*[tiab] OR "anti inflam*"[tiab] OR anti-inflam*[tiab] OR antiinflam*[tiab] OR cytokine*[tiab] OR “C-reactive protein”[tiab] OR “C reactive protein”[tiab] OR CRP[tiab] OR hsCRP[tiab] OR hs-CRP[tiab] OR “hs CRP” [tiab] OR “high-sensitivity-C reactive protein” [tiab] OR “high-sensitivity-C-reactive protein” [tiab] OR “high sensitivity-C-reactive protein” [tiab] OR “high sensitivity-C reactive protein” [tiab] OR “high sensitivity-CRP”[tiab] OR high-sensitivity-CRP[tiab] OR “high-sensitive-C reactive protein”[tiab] OR “high-sensitive-C-reactive protein”[tiab] OR “high sensitive-C-reactive protein”[tiab] OR “high sensitive-C reactive protein”[tiab] OR “high sensitive-CRP”[tiab] OR high-sensitive-CRP[tiab] OR Interleukin*[tiab] OR “Interleukin 6” [tiab] OR Interleukin-6[tiab] OR IL[tiab] OR “IL 6”[tiab] OR IL6[tiab] OR IL-6[tiab] OR “Interferon beta-2”[tiab] OR “Interferon beta 2”[tiab] OR “Interferon beta2”[tiab] OR “Interferon β2”[tiab] OR “Interferon β 2”[tiab] OR “IFN-beta 2”[tiab] OR IFN-beta2[tiab] OR “IFN beta2”[tiab] OR “IFN beta-2”[tiab] OR “IFN beta 2”[tiab] OR IFN-β2[tiab] OR “IFN-β 2”[tiab] OR “IFN β2”[tiab] OR “Tumor Necrosis Factor*”[tiab] OR “Tumor Necrosis Factor-alpha”[tiab] OR “Tumor Necrosis Factor-α”[tiab] OR “Tumor Necrosis Factor alpha”[tiab] OR “Tumor Necrosis Factor α”[tiab] OR TNF[tiab] OR TNFs[tiab] OR TNF-alpha[tiab] OR TNF-α[tiab] OR TNFalpha[tiab] OR TNFα[tiab] OR “TNF alpha”[tiab] OR “TNF α”[tiab] OR TNFa[tiab] OR TNF-a[tiab] OR cachectin*[tiab] OR cachexin*[tiab] OR “Cachectin-Tumor Necrosis Factor*”[tiab] OR Cachectin-TNF*[tiab]) AND 1990/01/01:2021/09/23[dp]

**Web of Science:**

(TS=(whey*) OR TS=("whey protein*") OR TS=("milk protein*") OR TS=("dairy protein*")) AND (TS=(inflam*) OR TS=(“pro inflam*”) OR TS=(pro-inflam*) OR TS=(proinflam*) OR TS=("anti inflam*") OR TS=(anti-inflam*) OR TS=(antiinflam*) OR TS=(cytokine*) OR TS=(“C-reactive protein”) OR TS=(“C reactive protein”) OR TS=(CRP) OR TS=(hsCRP) OR TS=(hs-CRP) OR TS=(“hs CRP”) OR TS=(“high-sensitivity-C reactive protein”) OR TS=(“high-sensitivity-C-reactive protein”) OR TS=(“high sensitivity-C-reactive protein”) OR TS=(“high sensitivity-C reactive protein”) OR TS=(“high sensitivity-CRP”) OR TS=(high-sensitivity-CRP) OR TS=(“high-sensitive-C reactive protein”) OR TS=(“high-sensitive-C-reactive protein”) OR TS=(“high sensitive-C-reactive protein”) OR TS=(“high sensitive-C reactive protein”) OR TS=(“high sensitive-CRP”) OR TS=(high-sensitive-CRP) OR TS=(interleukin*) OR TS=(“interleukin 6”) OR TS=(interleukin-6) OR TS=(IL) OR TS=(IL-*) OR TS=(“IL 6”) OR TS=(IL6) OR TS=(IL-6) OR TS=(“interferon beta-2”) OR TS=(“interferon beta 2”) OR TS=(“interferon beta2”) OR TS=(“interferon β2”) OR TS=(“interferon β 2”) OR TS=(“IFN-beta 2”) OR TS=(IFN-beta2) OR TS=(“IFN beta2”) OR TS=(“IFN beta-2”) OR TS=(IFN-β2) OR TS=(“IFN-β 2”) OR TS=(“IFN β2”) OR TS=(“IFN beta 2”) OR TS=(“tumor necrosis factor*”) OR TS=(“tumor necrosis factor-alpha”) OR TS=(“tumor necrosis factor-α”) OR TS=(“tumor necrosis factor alpha”) OR TS=(“tumor necrosis factor α”) OR TS=(TNF*) OR TS=(TNF-alpha) OR TS=(TNF-α) OR TS=(TNFalpha) OR TS=(TNFα) OR TS=(“TNF alpha”) OR TS=(“TNF α”) OR TS=(TNFa) OR TS=(TNF-a) OR TS=(cachectin*) OR TS=(cachexin*) OR TS=(“cachectin-tumor necrosis factor*”) OR TS=(cachectin-TNF*)) AND PY=(1990-2021)

**Scopus**:

(TITLE-ABS-KEY(whey*) OR TITLE-ABS-KEY("whey protein*") OR TITLE-ABS-KEY("milk protein*") OR TITLE-ABS-KEY("dairy protein*")) AND (TITLE-ABS-KEY(inflam*) OR TITLE-ABS-KEY(“pro inflam*”) OR TITLE-ABS-KEY(pro-inflam*) OR TITLE-ABS-KEY(proinflam*) OR TITLE-ABS-KEY("anti inflam*") OR TITLE-ABS-KEY(anti-inflam*) OR TITLE-ABS-KEY(antiinflam*) OR TITLE-ABS-KEY(cytokine*) OR TITLE-ABS-KEY(“C-reactive protein”) OR TITLE-ABS-KEY(“C reactive protein”) OR TITLE-ABS-KEY(CRP) OR TITLE-ABS-KEY(hsCRP) OR TITLE-ABS-KEY(hs-CRP) OR TITLE-ABS-KEY(“hs CRP”) OR TITLE-ABS-KEY(“high-sensitivity-C reactive protein”) OR TITLE-ABS-KEY(“high-sensitivity-C-reactive protein”) OR TITLE-ABS-KEY(“high sensitivity-C-reactive protein”) OR TITLE-ABS-KEY(“high sensitivity-C reactive protein”) OR TITLE-ABS-KEY(“high sensitivity-CRP”) OR TITLE-ABS-KEY(high-sensitivity-CRP) OR TITLE-ABS-KEY(“high-sensitive-C reactive protein”) OR TITLE-ABS-KEY(“high-sensitive-C-reactive protein”) OR TITLE-ABS-KEY(“high sensitive-C-reactive protein”) OR TITLE-ABS-KEY(“high sensitive-C reactive protein”) OR TITLE-ABS-KEY(“high sensitive-CRP”) OR TITLE-ABS-KEY(high-sensitive-CRP) OR TITLE-ABS-KEY(interleukin*) OR TITLE-ABS-KEY(“interleukin 6”) OR TITLE-ABS-KEY(interleukin-6) OR TITLE-ABS-KEY(IL) OR TITLE-ABS-KEY(IL-*) OR TITLE-ABS-KEY(“IL 6”) OR TITLE-ABS-KEY(IL6) OR TITLE-ABS-KEY(IL-6) OR TITLE-ABS-KEY(“interferon beta-2”) OR TITLE-ABS-KEY(“interferon beta 2”) OR TITLE-ABS-KEY(“interferon beta2”) OR TITLE-ABS-KEY(“interferon β2”) OR TITLE-ABS-KEY(“interferon β 2”) OR TITLE-ABS-KEY(“IFN-beta 2”) OR TITLE-ABS-KEY(IFN-beta2) OR TITLE-ABS-KEY(“IFN beta2”) OR TITLE-ABS-KEY(“IFN beta-2”) OR TITLE-ABS-KEY(IFN-β2) OR TITLE-ABS-KEY(“IFN-β 2”) OR TITLE-ABS-KEY(“IFN β2”) OR TITLE-ABS-KEY(“IFN beta 2”) OR TITLE-ABS-KEY(“tumor necrosis factor*”) OR TITLE-ABS-KEY(“tumor necrosis factor-alpha”) OR TITLE-ABS-KEY(“tumor necrosis factor-α”) OR TITLE-ABS-KEY(“tumor necrosis factor alpha”) OR TITLE-ABS-KEY(“tumor necrosis factor α”) OR TITLE-ABS-KEY(TNF*) OR TITLE-ABS-KEY(TNF-alpha) OR TITLE-ABS-KEY(TNF-α) OR TITLE-ABS-KEY(TNFalpha) OR TITLE-ABS-KEY(TNFα) OR TITLE-ABS-KEY(“TNF alpha”) OR TITLE-ABS-KEY(“TNF α”) OR TITLE-ABS-KEY(TNFa) OR TITLE-ABS-KEY(TNF-a) OR TITLE-ABS-KEY(cachectin*) OR TITLE-ABS-KEY(cachexin*) OR TITLE-ABS-KEY(“cachectin-tumor necrosis factor*”) OR TITLE-ABS-KEY(cachectin-TNF*)) AND ((PUBYEAR > 1989 AND PUBYEAR < 2021) OR PUBDATETXT("January 2021" OR "February 2021" OR "March 2021" OR "April 2021" OR "May 2021" OR "June 2021" OR "July 2021" OR "August 2021" OR "September 2021"))
