# Supplementary material for: Acute or Short-term Effects of Whey Protein Alone or Along with Carbohydrate on Inflammation: A Systematic Review of Clinical Trials: -
Source: Galen Med J. 2023 Apr 18;12:e2441. doi: 10.31661/gmj.v12i.2441 (PMC10506851; doi:10.31661/gmj.v12i.2441)
Supplement: Supplementary file 2 [file GMJ-12-e2441-s2.docx]

**Supplementary file 2**

**Supplementary table 1**. Quality assessments of studies evaluating the acute or short term effects of whey protein on inflammatory markers

| **First author, year** | **Random sequence generation** | **Allocation concealment** | **Selective reporting** | **Other bias** | | **Blinding of participants and personnel** | **Blinding of outcome assessment** | **Incomplete outcome data** | **Overall quality** |
| --- | --- | --- | --- | --- | --- | --- | --- | --- | --- |
| Mizubuti, 2021 | Low risk | Unclear | Unclear | | Low risk | Low risk | Low risk | Low risk | Fair |
| Hilkens, 2021 | Low risk | Low risk | Unclear | | High risk | Low risk | Low risk | Low risk | Poor |
| Nieman, 2020 | Low risk | Unclear | High risk | | Unclear | Low risk | Low risk | Unclear | Poor |
| Saracino, 2020 | Unclear | Unclear | Unclear | | High risk | Unclear | Low risk | Low risk | Poor |
| Celik, 2019 | Unclear | Unclear | Unclear | | Unclear | Unclear | Low risk | Unclear | Poor |
| Snipe, 2017 | Unclear | Unclear | Unclear | | High risk | High risk | Low risk | Unclear | Poor |
| Mariotti, 2015 | Low risk | Unclear | Unclear | | Unclear | High risk | Low risk | Low risk | Poor |
| Schroer, 2014 | Unclear | Unclear | Unclear | | Low risk | Low risk | Low risk | Low risk | Poor |
| Kinsey, 2014 | Unclear | Unclear | Unclear | | Unclear | Low risk | Low risk | Low risk | Poor |
| Baba, 2014 | Unclear | Unclear | Unclear | | Low risk | Unclear | Low risk | Low risk | Poor |
| Singh, 2014 | Low risk | Low risk | Low risk | | Low risk | Unclear | Low risk | Low risk | Good |
| Pal, 2011 | Unclear | Unclear | High risk | | Low risk | Unclear | Low risk | Low risk | Poor |
| de Aguilar-Nascimento, 2011 | Low risk | Low risk | Unclear | | Unclear | Low risk | Low risk | Low risk | Fair |
| Kullisaar, 2011 | Unclear | Unclear | Unclear | | Unclear | Low risk | Low risk | Low risk | Poor |
| Buckley, 2010 | Unclear | Unclear | Unclear | | Unclear | Low risk | Low risk | Low risk | Poor |

**Supplementary table 2**. Quality assessments of studies evaluating the acute or short term effects of whey protein in combination with carbohydrate on inflammatory markers

| **First author, year** | **Random sequence generation** | **Allocation concealment** | **Selective reporting** | **Other bias** | | **Blinding of participants and personnel** | **Blinding of outcome assessment** | **Incomplete outcome data** | **Overall quality** |
| --- | --- | --- | --- | --- | --- | --- | --- | --- | --- |
| de Carvalho, 2021 | Low risk | Unclear | Unclear | | High risk | High risk | Low risk | Low risk | Poor |
| Deng, 2020 | Unclear | Unclear | Unclear | | Low risk | Low risk | Low risk | Low risk | Poor |
| Yi, 2020 | Low risk | Low risk | Low risk | | Low risk | High risk | Low risk | Low risk | Fair |
| Isenmann, 2019 | Unclear | Unclear | High risk | | Unclear | Unclear | Low risk | Unclear | Poor |
| Qin, 2019 | Unclear | Unclear | Unclear | | Unclear | Low risk | Low risk | Low risk | Poor |
| Qin, 2017 | High risk | Unclear | Unclear | | Unclear | Low risk | Low risk | Unclear | Poor |
| Dahlquist, 2017 | Unclear | Unclear | Unclear | | Low risk | High risk | Low risk | Low risk | Poor |
| Hansen, 2015 | Low risk | Unclear | Unclear | | High risk | High risk | Low risk | Unclear | Poor |
| Kerasioti, 2013 | Unclear | Unclear | Unclear | | Low risk | Low risk | Low risk | Unclear | Poor |
| Betts, 2009 | Unclear | Unclear | Unclear | | High risk | High risk | Low risk | Unclear | Poor |
